# Impact of mother’s own milk expression practices and processing treatments on infant health and growth outcomes: a systematic review protocol

**DOI:** 10.1136/bmjopen-2024-087539

**Published:** 2025-02-11

**Authors:** Serena Gandino, Marzia Giribaldi, Carolyn Smith, Tanya Cassidy, Laura Cavallarin, Karolina Karcz, Chiara Peila, Daniel Klotz, Agnieszka Bzikowska-Jura, Bartłomiej Walczak, Aleksandra Wesolowska

**Affiliations:** 1 Nuffield Department of Women's & Reproductive Health, University of Oxford, Oxford, UK; 2 Department of Public Health and Pediatrics, University of Turin, Torino, Piemonte, Italy; 3 Institute of Sciences of Food Productions, National Research Council, Grugliasco, Italy; 4 Bodleian Libraries, Oxford University, Banbury, UK; 5 Kathleen Lonsdale Institute for Human Health Research, Maynooth University, Maynooth, Ireland; 6 Department and Clinic of Neonatology, Wroclaw Medical University, Wroclaw, Poland; 7 Bethel Center of Pediatrics, Department of Neonatology and Pediatric Intensive Care Medicine, University Hospital OWL of Bielefeld University, Bielefeld, Nordrhein-Westfalen, Germany; 8 Laboratory of Human Milk and Lactation Research, Medical University of Warsaw, Warszawa, Mazowieckie, Poland; 9 Institute of Applied Social Sciences, University of Warsaw, Warszawa, Poland

**Keywords:** NEONATOLOGY, NUTRITION & DIETETICS, INFECTIOUS DISEASES, PUBLIC HEALTH

## Abstract

**Abstract:**

**Introduction:**

Breastfeeding is the biological norm for infant nutrition. In certain scenarios, feeding at the breast is not possible, because of either maternal or neonatal reasons. In those cases, infants can still receive expressed mother’s own milk (MOM) and its beneficial properties. Mothers can express their milk using a variety of methods, while applying different hygiene practices, in different settings; moreover, expressed milk might receive processing before it is fed to the infant, particularly to reduce transmission of viruses such as cytomegalovirus to premature infants. The present protocol was designed to gather the evidence on the effect that the expression method, the hygiene regimen and setting and any processing used on expressed MOM can have on the clinical outcomes of recipient infants.

**Methods:**

This systematic review will follow the methodological recommendations of the Cochrane Collaboration, in accordance with WHO recommendations and Preferred Reporting Items for Systematic Reviews and Meta-Analyses guidelines. We will apply a hybrid search strategy, by combining structured database search with predefined snowballing searches. We will include primary research studies only, without restrictions on the type and including observational studies; no publication time, setting or language restriction will be applied, provided the abstract is available in English. Studies evaluating different methods of MOM expression, hygiene practices or settings during expression, processing of MOM and reporting clinical outcomes on recipient infants will be eligible. The searches have been planned to be performed in April–May 2024. Two reviewers will independently perform the reference screening, data extraction and risk of bias analysis of eligible studies, by using standardised tools specific for each study design. Quantitative and thematic narrative data synthesis will be performed and statistical heterogeneity between studies tested. Meta-analyses of extracted data will be performed where applicable. For relevant outcomes, certainty of the evidence will be tested by using the Grading of Recommendations Assessment, Development and Evaluation approach.

**Ethics and dissemination:**

Ethical approval is not required for this study since no original data will be collected. There is patient and public involvement throughout this research process. The results of this review will be disseminated through publication in a peer-reviewed journal and through conference presentations. Moreover, this systematic review will inform recommendations on milk banking of the WHO Department of Nutrition and Food Safety.

**PROSPERO registration number:**

CRD42024523299.

**STRENGTHS AND LIMITATIONS OF THIS STUDY**

- Our search strategy involves an experienced information specialist and a team of researchers with wide experience in human milk science and services.
- Our search strategy is built to a high standard using Cochrane Systematic Review methodology.
- A hybrid search strategy will be used and run over many databases and trial repositories in order to capture the highest number of relevant studies.
- Because of the extensive body of evidence on human milk, clinical outcomes of interest will be included in the search, and limitations to major headings will be applied.
- To mitigate those limitations, a robust search strategy will be developed, which includes comprehensive variations of all the search terms.

## Introduction

 Breastfeeding is the biological norm for infants.^1^ Extensive evidence has shown that breastfed infants are less likely to die or develop infections, gastrointestinal and respiratory diseases in early life.^2^ Additionally, prolonged breastfeeding confers enduring advantages, such as diminished likelihood of asthma, diabetes, obesity, cardiovascular afflictions and enhanced neurodevelopmental outcomes.^3^,^6^

The WHO and the United Nations International Children’s Emergency Fund recommend that all infants should be fed by exclusive breastfeeding from birth to 6 months of age and to be continued with appropriate complementary food up to 2 years.^7^ In certain scenarios, feeding at the breast is not possible because of either neonatal or maternal reasons. In such instances, babies can still be fed their mothers' own milk (MOM), expressed and provided to the infant via a bottle, cup or similar methods. A mother may feed her infant with her expressed MOM because her baby is unable to feed effectively at the breast (as in prematurity or in other health conditions), because of separation from her baby, because of breast engorgement, to boost her milk supply or because of her own preference. In some specific cases, guidelines recommend using treated MOM to reduce the risk of viral infection (human T-cell lymphotropic virus; cytomegalovirus (CMV); varicella-zoster virus.^8^ This is particularly relevant in the case of very low birth weight and very preterm infants to reduce the risk of transmitting CMV, which can cause severe sepsis in this vulnerable population.^9^,^11^ Moreover, in case of HIV positive mothers, studies have evaluated heat treatments of MOM to reduce the risk of transmitting the virus through the milk.^12^

Mothers can express milk using a number of methods; moreover, different hygiene practices can be applied during milk expression, which can occur in different settings. Finally, expressed milk might receive, in specific clinical conditions, heat treatments before it is fed to the infant. All those variables may influence milk composition and may potentially impact on the health and growth outcomes of recipient infants.

### Description of the interventions

#### Methods of milk expression

There is a wide variety of methods to express milk. The one which is universally available is hand expression, where milk ejection is stimulated by hand compression of the breast. Another widespread option is the hot jar method, in which a glass jar, warmed up with hot water, has its base being cooled with a cold cloth; the temperature gradient creates a vacuum effect which promotes milk ejection. Finally, milk can be expressed through a pump, which creates a negative pressure that stimulates milk flow. Pumps can be either manual, battery or electric.

Becker *et al* in 2016 reviewed the available evidence on the effectiveness, safety and effect on milk composition of different methods of milk expression.^13^ Authors found no difference regarding the contamination of human milk between the different methods of expression. Regarding the effect on milk composition, milk expressed via hand expression or large electric pump was found to have a higher protein content than milk expressed by manual pumps, which could be beneficial for promoting infants’ growth, especially those born preterm.^14^ Hand expression was also associated with a higher sodium content, which could contribute to the recovery of the sodium deficiency that typically affects preterm infants, impairing their growth. Fat content was higher when expression was accompanied by breast massage. Milk energy content was similar between the different expression methods.^13^

#### Hygiene practices and settings during milk expression

Human milk can get contaminated with exogenous bacteria during expression and handling. Those bacteria may potentially cause sepsis in recipient infants, especially those born prematurely.^15^ Also, the setting of expression of MOM might have an influence, as some reports have revealed a higher contamination in samples expressed at home with respect to those expressed in the hospital.^16^ Again, whereby a higher contamination might not pose significant risks for healthy term infants, in specific clinical situations, this should be carefully evaluated.^17^ Hence, appropriate hygiene practices need to be applied to ensure milk safety.^17^ Mothers’ hand hygiene can be achieved by washing hands with soapy water or with an alcohol-based sanitiser. Breast cleansing can be performed with water only, water and soap or antibacterial wipes. Disinfectants can be used to clean the pumping area. When using a pump, pump parts that come into contact with the breast can be rinsed before expressing; after pumping, the kit can be cleaned and disinfected either by hand with warm soapy water, by using a dishwasher or by boiling the equipment. In the proposed review, we aim to collect evidence about the influence of different hygiene practices and different settings of expression on the health and growth outcomes of recipient infants.

#### Processing of expressed milk

Human milk is not sterile but contains a number of micro-organisms (the so-called ‘human milk microbiota’) that play an essential role in shaping the infant microbiome. It may, also, serve as a vector for infectious diseases and, therefore, might be subjected to different kinds of heat treatments to reduce the risk of transmission. Risks of transmission must be outweighed by the deleterious effect of any kind of treatment on the immunological and nutritional properties of MOM.^18^

Storage at different cold temperatures and duration has been used traditionally for MOM, in order to both preserve it for future use and to decrease/eliminate specific viral pathogens.^8^ Low temperature storage is also effective in preventing bacteria proliferation, though may negatively affect the immunological and nutritional properties of human milk.^18^ Pasteurisation of MOM can also be used. Among the techniques, Holder pasteurisation represents the best compromise currently available, offering safety but impairing milk quality which may influence health and growth outcomes of recipient infants.^19 20^ Recently, high temperature short time treatments of human milk have been introduced in clinical practice.^21^ Further technologies are being evaluated aiming to provide microbial safety while maintaining human milk quality to the utmost degree possible.^20 22^ The proposed review will assess the available evidence for the impact of thermal (freezing or heating milk, applying different time and temperature parameters) and non-thermal (eg, high pressure processing, sonication and UV-treatment) antimicrobial treatment of MOM on the health and growth outcomes of recipient infants.

### Why it is important to do this review?

Milk expression enables infants who cannot feed directly at the breast to still receive the beneficial properties of maternal milk. It also allows decontaminating MOM in specific clinical situations (viral infections and bacterial contamination), thus ensuring a compromise between the unique advantages of maternal milk and the safety of the infant. However, different expression practices, hygiene regimens and heat treatments may affect the composition of MOM, eventually influencing the health and growth outcomes of recipient infants. The present review protocol is designed to collect the available evidence on the effect that these processes can have on the clinical outcomes of the recipient offspring, including growth, neurodevelopment, morbidity, mortality, tolerance, adverse events/effects, infections, nutritional deficiencies and other health-related outcomes.

### Objectives

To examine the impact of feeding expressed MOM using varying expression practices or treatments on health, growth and developmental outcomes of recipient infants.

Particularly, the following questions will be addressed:

- Are infants receiving expressed MOM (*P*) differently affected in their growth, development and/or health status (*O*) when maternal milk is expressed with different methods (*I*_*1*_)?
- Are infants receiving expressed MOM (*P*) differently affected in their growth, development and/or health status (*O*) when different hygiene practices or settings are used during maternal milk expression (*I*_*2*_)?
- Are infants receiving expressed MOM (*P*) differently affected in their growth, development and/or health status (*O*) when maternal milk is treated with different methods (*I*_*3*_)?

## Methods and analysis

### Eligibility criteria

Published or unpublished primary research studies are eligible for inclusion. These include observational studies, cohort studies, case control studies and clinical trials. We will not impose any restriction on the language of the report, provided that the abstract is available in English. Translations of non-English manuscripts will be performed using large language models. We will not apply setting or time frame restrictions either.

We will exclude papers with no original data—including reviews, editorials, comments, letters, case studies and book chapters. However, the reference list of relevant reviews will be screened manually to identify additional potentially eligible studies. We will exclude conference proceedings and animal studies.

Whether unpublished or partially published results might be eligible, the authors will contact the author/researcher/contact person by email, who will be asked to integrate, when possible, available information on extracted data.

### Types of participants, interventions, and outcomes

The population of interest for the present review protocol is represented by infants, either born at term or preterm, receiving expressed MOM, either alone or in combination with breastfeeding, donor human milk (DHM) or formula.

The interventions of interest are represented by expression methods (eg, hand expression, different pump models and expression regimen); hygiene practices and settings (eg, sanitising protocol and/or washing procedure and expression at home or in hospital); thermal and/or non-thermal processing (eg, freezing and pasteurisation) applied to MOM.

No prespecified comparison group will be used in the search. However, the studies will not be considered eligible when comparison is made for infants receiving solely formula versus solely MOM or for infants receiving solely DHM versus solely MOM.

Health outcomes of interest will include growth outcomes; mortality; morbidity (which will include bronchopulmonary dysplasia, necrotising enterocolitis, retinopathy of prematurity, intraventricular haemorrhage and periventricular leucomalacia); feeding tolerance (defined by duration of parenteral nutrition, time to full enteral feeding or predefined feeding intolerance score); adverse events (defined as an undesired effect of the intervention under evaluation); CMV infection; retroviral infection; other infections (ie, bacterial infections, fungal infections, viral infections other than CMV and retroviruses); nutrient deficiencies; neurodevelopment (assessed through a standardised neurodevelopment assessment); breastfeeding rate; length of hospital stay; other clinical outcomes. Because of the foreseen paucity of included studies, we will not set in advance the timepoints for outcome assessment.

In recognition of the intentions of the WHO Guideline Development Group (GDG) regarding the original question, no prioritisation of either intervention type or outcomes will be performed. To ensure as broad and accurate discussion of available data related to this question, we will include studies reporting a combination of interventions.

### Information sources

The search will include the following electronic databases: Cochrane Central Register of Controlled Trials (CENTRAL), Global Index Medicus, Cumulative Index to Nursing and Allied Health Literature (CINAHL) on EBSCOhost, Medline, Embase, EmCare, PubMed, Global Health on OVID, Google Scholar, Scopus and Web of Science Core Collection.

We will search clinical trial registries for recently completed trials: CENTRAL, clinicaltrials.gov, the WHO International Trials Registry and Platform and the European Union (EU)/European Economic Area (EEA) Clinical Trials Register. No date restrictions will be applied.

### Search strategy

The search strategy has been designed by the librarian (CS) and reviewed and approved by the team. We will apply a hybrid search strategy, by combining structured database searches with snowballing searches, in order to identify the highest number of relevant primary studies.^23^ Regarding the database searches, the search strategy will consist of search words on the key concepts of ‘mother’s own milk’, ‘expressed or stored’ and ‘effect on infants’. The searches have been planned to be performed in April–May 2024.

The draft search strategy and permalink for MEDLINE are available in online supplemental appendix 1. These search terms will be adapted for use with the other bibliographic databases. The search strategies for each database are reported in online supplemental file 1.

The reference list of relevant reviews will be screened manually to identify additional potentially relevant studies. We will hand search PubMed and Google Scholar for reference lists, cited by and similar articles.^24^ This part of the search will be restricted to the 10 most cited articles we find in the main search and the first 200 records found in this way.

### Data management

This systematic review will follow the methodological recommendations of the Cochrane Collaboration,^24^ in accordance with WHO recommendations and Preferred Reporting Items for Systematic Reviews and Meta-Analyses (PRISMA) guidelines.^25^ The PRISMA for systematic review protocols (PRISMA-P) was used to prepare this protocol.^26^ The raw data collected are publications containing no sensitive data and available in text formats (pdf, docx, rtf, etc). The papers selected for a full-text review will be stored in COVIDENCE system. Additional backups will be stored on the team’s shared drive and offline. The data extracted with COVIDENCE (xls) and further transformed into sav format will be backed in the same way. The backups will be managed by one appointed team member.

### Selection process

The systematic literature search on selected databases will be conducted by the librarian (CS). The search results from different databases will be merged using COVIDENCE software, and duplicates will be removed by the software. Each article will be independently screened by two authors with previous experience in the field of human milk science. This screening stage will use titles and abstracts to remove irrelevant reports on the basis of eligibility and inclusion criteria. When uncertainty about inclusion of the study arises, the study paper will proceed to the full-text screening stage. Once the initial Title and Abstract screening is complete, all remaining studies will be retrieved and independently screened in full text by two authors. A third review author will be involved at the end of this stage, to resolve conflicts.

### Data collection process

A standardised data extraction form will be used to report study characteristics, participants, interventions and outcomes, in the online software COVIDENCE. The data extraction sheet is provided in online supplemental appendix 2.

Two review authors will independently extract the data of interest from the included studies and evaluate the study’s quality using the online forms. The extracted data will be cross-checked and, if any difference will be identified, the full review team will discuss the decision until it is resolved.

If study data are only available from figures, data will be extracted by use of the validated software PlotDigitizer (PlotDigitizer). Missing or uncertain data will be handled by tentative contact with the study investigator by email for data or additional details. The same approach will be followed for ongoing studies and clinical trials. Data extracted with COVIDENCE will be transferred to sav format for further statistical analysis with SPSS software, version 29.0.2.0.

### Risk of bias in individual studies

Two reviewers will assess each included study for risk of bias (RoB), independently. In case of disagreement, the full review team will be asked for consultation. All eligible studies will be formally evaluated using standardised tools. Critical Appraisal Skills Programme (CASP, 2023) and RoB indicators will be included in the quality control form. For randomised controlled trials studies, the RoB will be assessed by using RoB 2.^24^ For non-randomised controlled trials, the Risk Of Bias In Non-randomised Studies - of Interventions tool.^27^ For observational studies, the Newcastle Ottawa Scale will be considered.^28^

### Data synthesis

Two methods of synthesis will be provided. For quantitative data, we will group similar data and present the results (distribution, mean, SD and effect size when available) in tables; we will provide graphical presentation—forest plots, for example, for combining the results of multiple clinical trials to show point estimates arising from different studies concerning the impact of the expression method, treatment and storage of MOM over the growth and health outcomes of recipient infants. On the forest plots, mean values with CIs for each study will be provided. Additionally, each mean will be plotted relative to the vertical line of no difference. The analysis will be performed using Excel and SPSS software.

Subgroup analyses, due to the anticipated variability in interventions, both alone or in combination, will depend on the nature and quantity of data retrieved. In particular, the number of studies that contain information about the subgroup in question will primarily determine whether the subgroup analysis is feasible.

For appropriate studies, we will perform a thematic analysis—identifying and coding information about the selected studies’ methodologies and findings,^29^ for example, electric versus manual breast pumps; organising the codes into subheadings and descriptive categories; developing these categories into analytical themes. This analysis will be performed using MAXQDA software and will result in a systematically produced narrative summary.

In case of studies reporting a combination of interventions, this will be reported in a narrative analysis, and the outcomes will be extracted for each intervention, analysed and, if possible, included potential data synthesis. All decisions for the data synthesis will follow Chapter 10 from the Cochrane handbook about how to analyse data and best practices for meta-analyses.^24^

### Metabiases

We will conduct tests for statistical heterogeneity, as advocated in PRISMA 2020.^25^ We will evaluate variability between the studies involved in this review by using the Cochran Q and creating a forest plot representation of these data.

### Confidence in cumulative evidence

We will use the Grading of Recommendations, Assessment, Development and Evaluation (GRADE) approach, as outlined in the GRADE Handbook^30^ to assess the certainty of evidence for the clinically relevant outcomes determined by consensus by the clinical members of the team and specifically include growth (measured by weight, length and head circumference), mortality, morbidity, feeding tolerance, adverse events, infections, nutrient deficiencies and neurodevelopment. The specific GRADE comparisons will be determined based on the evidence we obtain in this review and will involve clinical as well as methodological input. Two review authors will independently assess the certainty of the evidence for each of the outcomes of interest.

The GRADE approach provides an assessment of the certainty of a body of evidence based on four grades (table 1).

**Table 1:** Four grades of certainty of evidence according to Grading of Recommendations Assessment, Development and Evaluation approach

High certainty	Further research is very unlikely to change our confidence in the estimate of effect.
Moderate certainty	Further research is likely to have an important impact on our confidence in the estimate of effect and may change the estimate.
Low certainty	Further research is very likely to have an important impact on our confidence in the estimate of effect and is likely to change the estimate.
Very low certainty	We are very uncertain about the estimate.

In the case of small sample size or small number of studies, we will consider the potential for publication bias and follow the GRADE guidelines specifically for rating quality of evidence—publication bias.^31^

## Ethics and dissemination

Ethical approval is not required for this study since no original data will be collected. The results of this review will be disseminated through publication in a peer-reviewed journal and through conference presentations. Moreover, this systematic review will inform recommendations on milk banking of the WHO Department of Nutrition and Food Safety.

### Patient and public involvement

The research question involved in this systematic review is provided by the WHO GDG. The WHO GDG is made up of members from diverse backgrounds, including users of services, which means that there is direct patient and public involvement (PPI) from the onset of this work and the development of the research question. The systematic review team also consists of people from diverse backgrounds and includes people who are current service users, as well as service users in the past, again indicating that PPI is integrated throughout this research, including design, data extraction and analysis, as well as dissemination. This diversity of backgrounds involved in the team is one of the strengths and reason why the WHO was keen to have the team complete this systematic review.

The Human Milk Bank Foundation (the guarantor of our WHO sponsored systematic review) is a patient organisation entered into the Polish register of the Patient Rights Ombudsman (RzPP-DWS.072.41.2023) representing the interests and needs of premature and sick children and their caregivers in the field of equal access to breast milk and the principles of financing nutritional therapy as part of the guaranteed benefit. The protocol consulted and will consult on an ongoing basis with representatives of the PPI organisation, including AW, representing the Human Milk Bank Foundation as the WHO’s contractor.

## supplementary material

10.1136/bmjopen-2024-087539 online supplemental file 1

10.1136/bmjopen-2024-087539 online supplemental file 2

10.1136/bmjopen-2024-087539 online supplemental file 3
