# Supplementary material for: Impact of mother’s own milk expression practices and processing treatments on infant health and growth outcomes: a systematic review protocol
Source: BMJ Open. 2025 Feb 11;15(2):e087539. doi: 10.1136/bmjopen-2024-087539 (PMC11815424; doi:10.1136/bmjopen-2024-087539)
Supplement: online supplemental file 2 [file bmjopen-15-2-s002.docx]

**Appendix I.** Search strategy for Medline

Permalink:

<https://ovidsp.ovid.com/ovidweb.cgi?T=JS&NEWS=N&PAGE=main&SHAREDSEARCHID=4hBcar3IM40CZutGVyvKqSFKS4kGQT0tNiuanYRXdxdyLZl4vweDNMCbZaDejBWm0>

Medline (Ovid MEDLINE® Epub Ahead of Print, In-Process & Other Non-Indexed Citations, Ovid MEDLINE® Daily and Ovid MEDLINE®) 1946 to present

| 1 | *Milk, Human/ or *Breast Feeding/ or exp *Lactation/ |
| --- | --- |
| 2 | (express* or pump* or extract* or collect* or treat* or pasteuri* or heat or thermal or composition or process* or microbial or antimicrobial or contaminat* or freez* or froze* or sonic* or UV or hygien* or save* or saving or stor* or keep* or conserv* or preserv* or Unpasteuri* or Steril* or microbiolog* or bacteria* or cool* or macronutrient*).ti,ab,kf. |
| 3 | 1 and 2 |
| 4 | ((breastmilk or ((breast or human or mother* or parent or parents) and milk) or breastfe* or breast feed* or breast fed or colostrum or lactat*) adj3 (express* or pump* or extract* or collect* or treat* or pasteuri* or heat or thermal or composition or process* or microbial or antimicrobial or contaminat* or freez* or froze* or sonic* or UV or hygien* or save* or saving or stor* or keep* or conserv* or preserv* or Unpasteuri* or Steril* or microbiolog* or bacteria* or cool* or macronutrient*)).ti,ab,kf. |
| 5 | Breast Milk Expression/ |
| 6 | hand express*.ti,ab,kf. |
| 7 | 3 or 4 or 5 or 6 |
| 8 | exp *Infant/ |
| 9 | (Outcome* or impact * or affect* or influenc* or result* or consequenc* or caus* or issue* or threat* or effect* or benefit* or harm* or risk factor* or tolera* or growth* or development* or neurodevelop* or behavio* or weight or digest* or diet or nutrition or malnutrition or deficien* or illness* or death* or morbidity or mortality or infection* or transmi* or sepsis* or enterocolitis* or cytomegalovirus* or HIV or immunologic or diarrh* or meningitis or urinary tract infection or UTI or Expos* or Quality or Complication*).ti,ab,kf. |
| 10 | 8 and 9 |
| 11 | ((infant* or baby or babies or neonate* or new born* or newborn* or preterm or pre-term or child*) adj4 (Outcome* or impact * or affect* or influenc* or result* or consequenc* or caus* or issue* or threat* or effect* or benefit* or harm* or risk factor* or tolera* or growth* or development* or neurodevelop* or behavio* or weight or digest* or diet or nutrition or malnutrition or deficien* or illness* or death* or morbidity or mortality or infection* or transmi* or sepsis* or enterocolitis* or cytomegalovirus* or HIV or immunologic or diarrh* or meningitis or urinary tract infection or UTI or Expos* or Quality or Complication*)).ti,ab,kf. |
| 12 | ((infant* or baby or babies or neonate* or new born* or newborn* or preterm or pre-term or child*).ti,ab,kf. or exp *Infant/) and exp *"Growth and Development"/ |
| 13 | 10 or 11 or 12 |
| 14 | 7 and 13 |
| 15 | 14 not (Animals/ not (Animals/ and Humans/)) |
