# Supplementary material for: Impact of mother’s own milk expression practices and processing treatments on infant health and growth outcomes: a systematic review protocol
Source: BMJ Open. 2025 Feb 11;15(2):e087539. doi: 10.1136/bmjopen-2024-087539 (PMC11815424; doi:10.1136/bmjopen-2024-087539)
Supplement: Supplementary file 4 [file bmjopen-2024-087539.reviewer_comments.pdf]

PEER REVIEW HISTORY

BMJ Open publishes all reviews undertaken for accepted manuscripts. Reviewers are asked to complete a checklist review form and are provided with free text boxes to elaborate on their assessment. These free text comments are reproduced below.

ARTICLE DETAILS

Title (Provisional)

The Impact of Mother's Own Milk Expression Practices and Processing Treatments on Infant Health and Growth Outcomes: a systematic review protocol

Authors

Gandino, Serena; Giribaldi, Marzia; Smith, Carolyn; Cassidy, Tanya; Cavallarin, Laura; Karcz, Karolina; Peila, Chiara; Klotz, Daniel; Bzikowska-Jura, Agnieszka; Walczak, Bartłomiej; Wesolowska, Aleksandra

---

VERSION 1 - REVIEW

**Reviewer**	**1**
**Name**	**Rumbold, Alice R.**
**Affiliation**	**South Australia Health, Women and Kids Theme**
**Date**	**10-May-2024**
**COI**	**I have received funding from the National Health and Medical Research Council in Australia for studies examining strategies to promote human milk feeding in preterm infants.**

The protocol describes the methods for an important review topic that will help guide recommendations about expression of breast milk to optimise infant health.

As it stands, there are a number of points that require clarification to ensure that the review methods are described sufficiently and address the research objectives. These are described and numbered below.

1. The background includes description of four main interventions – milk expression, hygiene practices, milk storage and milk treatment. However, in the abstract and objectives section there appear to be only three interventions. In the abstract it is expression, handling and treatment of MOM, and in the objectives section it is expression, hygiene practices and treatment. In addition, different 'settings' is introduced into research questions but not stated earlier. Please clarify the interventions that will be studied and ensure consistency throughout the manuscript.

2. The title should also be revised to include the focus on hygiene practices and storage.

3. Line 113 – include development here in addition to growth?

4. Lines 129-130 – the high protein content found with hand expression or electric pump – what is the comparison group here? Please include a reference to the statement about a higher protein content being beneficial for promoting preterm infant growth.

5. The background needs strengthening with additional references, particularly regarding the statements in lines 132-136, 140-141, 151-159, 168-170.

6. Lines 191-192 – include development outcomes as part of the overarching objective

7. The second research question focusses on ‘different hygiene practices or settings’. What is meant by settings e.g. hospital versus home? Or different country settings? The background could be strengthening by including a discussion of the potential impact of different settings to strengthen the rationale for this question.

8. It is not clear what types of study design will be included. Please specify in the eligibility criteria which types of study designs will be considered, and the comparison groups that will be considered to address each research question.

9. How will studies examining donor human milk will be treated, given there is a vast literature examining different treatment methods for banked donor human milk. Presumably these studies will be excluded? The protocol would be strengthened by briefly mentioning this in the background and including details of how donor milk studies will be considered.

10. The outcomes are poorly described (e.g. ‘morbidity’ and ‘growth outcomes’) and not defined. Please include definitions and times points, e.g. neurodevelopment at 2 years of age? Nutritional deficiencies – when? It would also be worth considering stating which are the critical outcomes and which are the important outcomes (as per Cochrane recommendations), or alternatively grouping these as primary and secondary outcomes.

11. Please specify which outcomes will be used in the GRADE assessment.

12. The abstract could be significantly strengthened, e.g. rephrasing that the review will ‘collect available evidence’, stating the key research questions that will be addressed, specifying which study designs will be eligible, what the outcomes are and that a meta-analysis will be performed where possible.

13. It is not clear how qualitative studies will contribute to this review – which outcomes will be assessed qualitatively, as they all appear to be clinical outcomes.

14. Will any subgroup analyses be considered, e.g. to see if the effects are different in preterm infants and term infants?

15. There are some grammatical and typographical errors, the manuscript would benefit from a thorough check for these. E.g. Line 188- DHM is mentioned, should this be MOM? Lines 67 & 346 – baking should be banking, line 72 – strengths is spelt incorrectly.

16. The limitations section is in the past tense – implies this has already been undertaken, however, this is a protocol.

Reviewer 2

Name Mildon, Alison

Affiliation University of Toronto Temerty Faculty of Medicine, Nutritional Sciences

Date 07-Aug-2024

COI I have no competing interests to declare.

Thank you for the opportunity to review this protocol for a systematic review on health and growth outcomes for infants receiving expressed mother's own milk. This is an important but complex topic area. There is a need to improve clarity in defining the Population, Interventions and Outcomes of focus for this review.

• Population: Please clarify whether the population of interest is infants receiving expressed milk from their own mother (suggested in lines 97-102 and by the term MOM) or infants receiving any expressed human milk (suggested by reference to DHM in line 188). If the population is restricted to infants receiving their own mother's expressed milk, this should be noted as a key criteria for the screening and selection of studies. Please also note whether any sub-groupings are planned for the synthesis and reporting of findings, such as preterm vs term born infants.

• Interventions: Lines 116-177 describe multiple practices related to human milk expression, storage and treatment. Will all of these be considered or are any of greater priority for this review? It would be clearer for the reader if the included Interventions were listed in the Methods, not the Rationale, as they are key to study eligibility and inclusion. Please also explain whether combinations of Interventions will be considered, and how this will be handled in data synthesis and reporting of findings.

• Outcomes: Definition of outcomes is missing in the abstract, which refers only to 'clinical impact' (line 51). Lines 290-294 list a variety of outcome categories but where possible, further specification and/or prioritization (with rationale) would be helpful. For example, which infections and illnesses are of particular interest? Please also move the text on Outcomes earlier in the Methods, as this is key to eligibility and inclusion of studies.

A few other areas for clarification:

• Eligibility: Please state which study designs will be eligible.

• Data collection: Please explain plans to refine and pretest the data extraction tool.

• Data synthesis: In addition to the tables and forest plots, will a narrative summary of findings be provided? Please add a comment on any plans related to meta-analysis, or the rationale for not including this. Synthesis of qualitative data is mentioned (lines 316-320) but it is not clear how qualitative studies would address the research questions.

• Please correct typos in line 70 (PROSPERO) and lines 67 and 346 (milk banking)

• Please provide a reference for the Newcastle-Ottawa Scale (line 304)

VERSION 1 - AUTHOR RESPONSE

- Reviewer: 1

Dr. Alice R. Rumbold, South Australia Health
Comments to the Author:
The protocol describes the methods for an important review topic that will help guide recommendations about expression of breast milk to optimise infant health. As it stands, there are a number of points that require clarification to ensure that the review methods are described sufficiently and address the research objectives. These are described and numbered below.

1. The background includes description of four main interventions – milk expression, hygiene practices, milk storage and milk treatment. However, in the abstract and objectives section there appear to be only three interventions. In the abstract it is expression, handling and treatment of MOM, and in the objectives section it is expression, hygiene practices and treatment. In addition, different 'settings' is introduced into research questions but not stated earlier. Please clarify the interventions that will be studied and ensure consistency throughout the manuscript.

Dear reviewer, thank you for this helpful comment. We reviewed our manuscript and adopted the description of three interventions (milk expression, hygiene practices and settings, thermal processing) throughout the text. Since storage regimens are still forms of thermal processing, they have been described together with heat treatments.

2. The title should also be revised to include the focus on hygiene practices and storage.

Dear reviewer, thank you for pointing this out. We decided to simplify our title, which has been changed to "The Impact of Mother's Own Milk Expression Practices and Processing Treatments on Infant Health and Growth Outcomes: a systematic review protocol".

3. Line 113 – include development here in addition to growth?

Thanks for your suggestion, which has been implemented accordingly.

4. Lines 129-130 – the high protein content found with hand expression or electric pump – what is the comparison group here? Please include a reference to the statement about a higher protein content being beneficial for promoting preterm infant growth.

Dear reviewer, thanks for highlighting this. We modified our manuscript by specifying the comparison group: milk expressed via hand expression or large electric pump versus milk expressed by manual pumps. We included a reference regarding the possible beneficial effect of a higher protein milk content for promoting preterm infant growth (Embleton, N. D., & van den Akker, C. H. P. (2019). Protein intakes to optimize outcomes for preterm infants. Seminars in perinatology, 43(7), 151154. https://doi.org/10.1053/j.semperi.2019.06.002).

5. The background needs strengthening with additional references, particularly regarding the statements in lines 132-136, 140-141, 151-159, 168-170.

Thanks for this comment. We strengthened our background section with additional references as you suggested.

6. Lines 191-192 – include development outcomes as part of the overarching objective

Thanks for this comment. We have implemented your suggestion.

7. The second research question focuses on 'different hygiene practices or settings'. What is meant by settings e.g. hospital versus home? Or different country settings? The background could be strengthening by including a discussion of the potential impact of different settings to strengthen the rationale for this question.

Thanks for this comment. We changed our manuscript by specifying what we meant by setting and how different settings can influence milk composition, and by adding related reference.

8. It is not clear what types of study design will be included. Please specify in the eligibility criteria which types of study designs will be considered, and the comparison groups that will be considered to address each research question.

Thanks for this comment. We implemented your suggestion and specified the types of study design that will be included in the "Eligibility criteria" subsection. Regarding comparison groups, we added specification of our strategy in the "PICO elements" subsection.

9. How will studies examining donor human milk will be treated, given there is a vast literature examining different treatment methods for banked donor human milk. Presumably these studies will be excluded? The protocol would be strengthened by briefly mentioning this in the background and including details of how donor milk studies will be considered.

Dear reviewer, thanks for this comment. We modified the "PICO elements" subsection by underlining that only studies evaluating Mother's Own Milk will be included.

10. The outcomes are poorly described (e.g. 'morbidity' and 'growth outcomes') and not defined. Please include definitions and times points, e.g. neurodevelopment at 2 years of age? Nutritional deficiencies – when? It would also be worth considering stating which are the critical outcomes and which are the important outcomes (as per Cochrane recommendations), or alternatively grouping these as primary and secondary outcomes.

Thanks for this comment. We welcomed your suggestion and expanded definitions of the outcomes in the "PICO elements" subsection. Because of the potential paucity of included studies, we have not set in advance timepoints for outcome assessment: this will allow us to be more broad and capture as much information as possible. Prioritisation of outcomes has been discussed with the WHO committee, who asked not to prioritise outcomes. Hence, prioritisation has not been performed. The order of the outcomes is in line with WHO call.

11. Please specify which outcomes will be used in the GRADE assessment.

Thank you for this comment. We have specified the clinical outcomes we will use for the review. And have also noted, that the specific GRADE comparisons will be determined based on the evidence and will involve clinical as well as methodological input.

12. The abstract could be significantly strengthened, e.g. rephrasing that the review will 'collect available evidence', stating the key research questions that will be addressed,

specifying which study designs will be eligible, what the outcomes are and that a meta-analysis will be performed where possible.

Thanks for this comment. We have implemented your suggestions.

13. It is not clear how qualitative studies will contribute to this review – which outcomes will be assessed qualitatively, as they all appear to be clinical outcomes.

Thank you for this comment. It is our intention to not restrict our data collection. We have made some additional edits in this section based on your comments.

14. Will any subgroup analyses be considered, e.g. to see if the effects are different in preterm infants and term infants?

Thanks for this comment. We added considerations on subgroup analyses in the "Data synthesis" subsection. Subgroup analyses, due to the anticipated variability in interventions, both alone or in combination, will depend on the nature and quantity of data retrieved. In particular, the number of studies that contain information about the subgroup in question will primarily determine whether the subgroup analysis is feasible.

15. There are some grammatical and typographical errors, the manuscript would benefit from a thorough check for these. E.g. Line 188- DHM is mentioned, should this be MOM?

Lines 67 & 346 – baking should be banking, line 72 – strengths is spelt incorrectly.

Thanks for highlighting those grammatical and typographical errors: we performed an extensive check and corrected them.

16. The limitations section is in the past tense – implies this has already been undertaken, however, this is a protocol.

Thanks for highlighting this: we modified the tense of the limitations section.

- Reviewer: 2
Dr. Alison Mildon, University of Toronto Temerty Faculty of Medicine

Comments to the Author: Thank you for the opportunity to review this protocol for a systematic review on health and growth outcomes for infants receiving expressed mother's own milk. This is an important but complex topic area. There is a need to improve clarity in defining the Population, Interventions and Outcomes of focus for this review.

• Population: Please clarify whether the population of interest is infants receiving expressed milk from their own mother (suggested in lines 97-102 and by the term MOM) or infants receiving any expressed human milk (suggested by reference to DHM in line 188). If the population is restricted to infants receiving their own mother's expressed milk, this should be noted as a key criterion for the screening and selection of studies. Please also note whether any sub-groupings are planned for the synthesis and reporting of findings, such as preterm vs term born infants.

Dear reviewer, thanks for this comment. We have added clarification of our population of interest in our "PICO elements" subsection: our population will be restricted to infants receiving Mother's Own Milk (MOM). Considerations regarding subgroup analysis have been added in the "Data synthesis" subsection.

• Interventions: Lines 116-177 describe multiple practices related to human milk expression, storage and treatment. Will all of these be considered or are any of greater priority for this review? It would be clearer for the reader if the included Interventions were listed in the Methods, not the Rationale, as they are key to study eligibility and inclusion. Please also explain whether combinations of Interventions will be considered, and how this will be handled in data synthesis and reporting of findings.

Dear reviewer, thanks for this comment. We modified our manuscript to improve clarity. Three interventions will be considered: expression method, hygiene practice and setting, thermal processing. These have been listed in the Methods, in the "PICO elements" subsection. In line with the WHO Guideline Development Group (GDG) who set the original question, no prioritisation of intervention type will be performed. This has been added in the "PICO elements" subsection. Also regarding your point about what we will do if confronted with combinations of interventions, we detailed that we will follow Chapter 10 from the Cochrane Handbook on best practice for analysing data and undertaking meta-analysis (this has been added in the "Data synthesis" subsection).

• Outcomes: Definition of outcomes is missing in the abstract, which refers only to 'clinical impact' (line 51). Lines 290-294 list a variety of outcome categories but where possible, further specification and/or prioritization (with rationale) would be helpful. For example, which infections and illnesses are of particular interest? Please also move the text on Outcomes earlier in the Methods, as this is key to eligibility and inclusion of studies.

Dear reviewer, thanks for highlighting this. We have added specification of the outcomes in the Abstract (in the "Introduction") and in the Methods (in the "PICO elements" subsection). Prioritisation of outcomes has been discussed with the WHO committee, who asked not to prioritise outcomes. Hence, prioritisation has not been performed.

A few other areas for clarification:

• Eligibility: Please state which study designs will be eligible.

Thanks for this comment. We have added specification of eligible study designs in the "Eligibility criteria" subsection.

• Data collection: Please explain plans to refine and pretest the data extraction tool.

Thanks for this comment. We have added this explanation in the "Data management" subsection.

• Data synthesis: In addition to the tables and forest plots, will a narrative summary of findings be provided?

Thank you for this comment. We have amended this section of the review with this comment in mind.

Please add a comment on any plans related to meta-analysis, or the rationale for not including this.

Thank you also for this comment, and we have amended this section of the protocol with this comment in mind.

Synthesis of qualitative data is mentioned (lines 316-320) but it is not clear how qualitative studies would address the research questions.

Thank you for this comment, which was also mentioned by the previous reviewer, and we have modified this section of the protocol to make this section clearer.

• Please correct typos in line 70 (PROSPERO) and lines 67 and 346 (milk banking)

Thanks for noticing these and they have been corrected.

• Please provide a reference for the Newcastle-Ottawa Scale (line 304)

Thank you and we have added a reference.

VERSION 2 - REVIEW

Reviewer **2**

Name **Mildon, Alison**

Affiliation **University of Toronto Temerty Faculty of Medicine, Nutritional Sciences**

Date **22-Nov-2024**

COI

Thank you for the thorough response to the previous review comments. The revisions have greatly improved the clarity of this protocol. I have no further comments on content, but please consider the following typographical edits when finalizing the manuscript:

• Lines 48, 108, 151: remove the word ‘thermal’ as the protocol includes non-thermal processing as well; add non-thermal in line 223

• Line 57, 257: report search dates in past tense

• Line 159 change ‘in order to extend its duration’ to ‘in order to preserve it for future use’

• Line 162 change first sentence wording to ‘Pasteurization of MOM can also be utilized’. Capitalize ‘Holder’.

• Line 224: change ‘Anyhow’ to “However”

• Line 228: define acronyms

VERSION 2 - AUTHOR RESPONSE

Reviewer: 2

Dr. Alison Mildon, University of Toronto Temerty Faculty of Medicine

Comments to the Author:

Thank you for the thorough response to the previous review comments. The revisions have greatly improved the clarity of this protocol. I have no further comments on content, but please consider the following typographical edits when finalizing the manuscript:

• Lines 48, 108, 151: remove the word ‘thermal’ as the protocol includes non-thermal processing as well; add non-thermal in line 223

Dear reviewer, thank you for this helpful comment. We revised our manuscript accordingly.

• Line 57, 257: report search dates in past tense

Dear reviewer, thank you for this comment. Search dates have now been reported in past tense.

• Line 159 change 'in order to extend its duration' to 'in order to preserve it for future use'

Dear reviewer, thank you for this suggestion, which has been implemented.

• Line 162 change first sentence wording to 'Pasteurization of MOM can also be utilized'. Capitalize 'Holder'.

Dear reviewer, thank you for these suggestions, which have been implemented.

• Line 224: change 'Anyhow' to "However"

Dear reviewer, thank you for this suggestion. We replaced "Anyhow" with "However".

• Line 228: define acronyms

Dear reviewer, thank you for highlighting this. Acronyms have been defined.

Reviewer: 2
If you have selected 'Yes' above, please provide details of any competing interests.: Not applicable
